# Predictive factors of postoperative complications in single-port video-assisted thoracoscopic anatomical resection: Two center experience

**DOI:** 10.1097/MD.0000000000012664

**Published:** 2018-10-05

**Authors:** Diego Gonzalez-Rivas, Yung Chia Kuo, Ching Yang Wu, Maria Delgado, de la Torre Mercedes, Ricardo Fernandez, Eva Fieira, Ming Ju Hsieh, Marina Paradela, Yin Kai Chao, Ching Feng Wu

**Affiliations:** a Coruña University Hospital; Minimally Invasive Thoracic Surgery Unit (UCTMI), Department of Thoracic Surgery, CORUÑA, Spain; b Chang Gung University, Division of Thoracic and Cardiovascular Surgery, Department of Surgery; c Chang Gung University, Division of Oncology, Department of Internal Medicine, Chang Gung Memorial Hospital, Linkou, Taiwan.

**Keywords:** anatomic resections, complications, single-port VATS

## Abstract

The purpose of this study was to identify the risk factors for adverse events during single-port video-assisted thoracoscopic (SPVATS) anatomical resections.

We retrospectively reviewed patients who had undergone SPVATS anatomic resections between January 2014 and February 2017 in Coruña University Hospital's Minimally Invasive Thoracic Surgery Unit (CHUAC, Spain) and Chang Gung Memorial Hospital (CGMH, Taiwan). Four hundred forty-two patients (male: 306, female: 136) were enrolled in this study. Logistic regression analysis was performed on variables for postoperative complications.

Postoperative complications with a 30-day mortality occurred in 94 patients (21.3%) and with a 90-day mortality in 3 patients (0.7%) while the major complication rate was 3.9%. Prolonged air leak (PAL > 5 days) was the most common complication and came by postoperative arrhythmia. Logistic regression indicated that pleural symphysis (odds ratio (OR), 1.91; 95% confidence interval (CI), 1.14–3.18; *P* = .014), computed tomography (CT) pulmonary emphysema (OR, 2.63; 95% CI, 1.41–4.76; *P* = .002), well-developed pulmonary CT fissure line (OR, 0.49; 95% CI, 0.29–0.84; *P* = .009), and tumor size (≥3 cm) (OR, 2.15; 95% CI, 1.30–3.57; *P* = .003) were predictors of postoperative complications.

Our preliminary results revealed that SPVATS anatomic resection achieves acceptable 30- and 90-day surgery related mortality (0.7%) and major complications rate (3.9%). Prolonged Air leak (PAL > 5 days) was the most common postoperative complication. Pleural symphysis, pulmonary emphysema, well-developed pulmonary CT fissure line and tumor size (≥3 cm) were predictors of adverse events during SPVATS anatomic resection.

## 1 Introduction

Surgery is a constantly evolving specialty. Since the era of thoracotomy, the evolutionary force has gradually driven forward minimally invasive surgery. Video-assisted thoracoscopic surgery (VATS) has been increasingly performed as an alternative to resection by thoracotomy since the first case was published 2 decades ago.^[1,2]^ The evolution and development of surgical facilities has helped surgeons to reduce the size and number of incision wounds. Single-port video-assisted thoracoscopic surgery (SPVATS) was recently selected as an option for the treatment of lung disease.^[3,4]^ In spite of potential advantages^[5–7]^ (less pain and shorter length of hospital stay), SPVATS presents the same challenges as other operation methods in development. Although it appears that SPVATS has comparable perioperative outcomes in numerous literatures,^[8–10]^ it is not clear whether postoperative complications such as prolonged air leaks >5 days (PAL), infection, and so on were relatively high and eventually outweighed previously mentioned potential benefit. This has led us to investigate 2 medical centers’ data in order to evaluate the morbidity of this relatively new technique. The object of this study has been twofold; one to completely describe the entire postoperative complications in SPVATS anatomic resections, the other to search for the predictors of complications.

## 2 Materials and methods

### 2.1 Patients

This is a retrospective observation study which enrolled patients undergoing single-port video-assisted thoracoscopic (SPVATS) anatomic resections in the Minimally Invasive Thoracic Surgery Unit at Coruña University Hospital (CHUAC, Spain) and Chang Gung Memorial Hospital (CGMH, Taiwan) from a prospectively maintained institutional thoracic database between January 2014 and February 2017. Study approval was obtained from the review board at Coruña University Hospital and Chang Gung Memorial Hospital, Linkou Branch (IRB No: 2013/092,20170805B0). Preoperatively, a series of examinations were arranged, including a pulmonary function test, chest plain film, chest and abdomen computed tomography (CT). As for patients with primary lung cancer, brain CT and positron emission tomography (PET) will be arranged. Where possible, cytological specimen or histological biopsy was obtained through bronchoscopy or CT-guided biopsy before surgery. Clinical data of each patient were prospectively collected and retrospectively analyzed.

### 2.2 Surgical techniques

Since the publish of the first case of SPVATS lobectomy in 2011,^[3]^ how to perform SPVATS surgery has gradually become widely known and standardized. The detailed operation methods were the same as we have described in previous literature.^[4,10]^ From January 2014, one self-taught consultant began to perform SPVATS in CGMH. To overcome the lack of relevant surgical experience, the consultant participated in a SPVATS training course in Shanghai Pulmonary Hospital (SPH, Shanghai). He gained competency and proficiency by following the steps of SPVATS major resection under Dr Gonzalez-Rivas's guidance in the SPH training course. After the trained consultant returned to Taiwan, he trained the other consultants in the same way.

Different types of anatomic resections were performed, depending on tumor size, location, and the resection margin determined from intraoperative frozen reports. A curative resection (R0 resection) was obtained for all enrolled patients. Segmentectomy was indicated for marginal pulmonary reserve patients or for those ground glass opacity lesion <2 cm without clinical evidence of hilar or mediastinal lymph node metastasis. All surgical procedures were performed under general anesthesia with the use of a double-lumen endotracheal tube. Enrolled patients received complete anatomic resections with systematic lymph node dissection in those with primary lung malignancy. One chest drainage tube was left for monitoring pleural effusion and air leakage. Criteria for discharge included: pleural effusion less than 250 mL/day, no air leakage, and absence of other complications.

### 2.3 Complications

Postoperative complications were all gathered and classified into a scale from I to V according to the definition of the Clavien-Dindo classification of surgical complications.^[11,12]^ Grades I and II stood for minor complications requiring no therapy or pharmacological intervention. Grades III and IV represented major complications requiring surgical intervention or life support. Grade V complications meant death. A detailed definition of the complications classification is listed in Table 1. If a patient had multiple concurrent complications, only the most severe complication was considered.

**Table 1:**
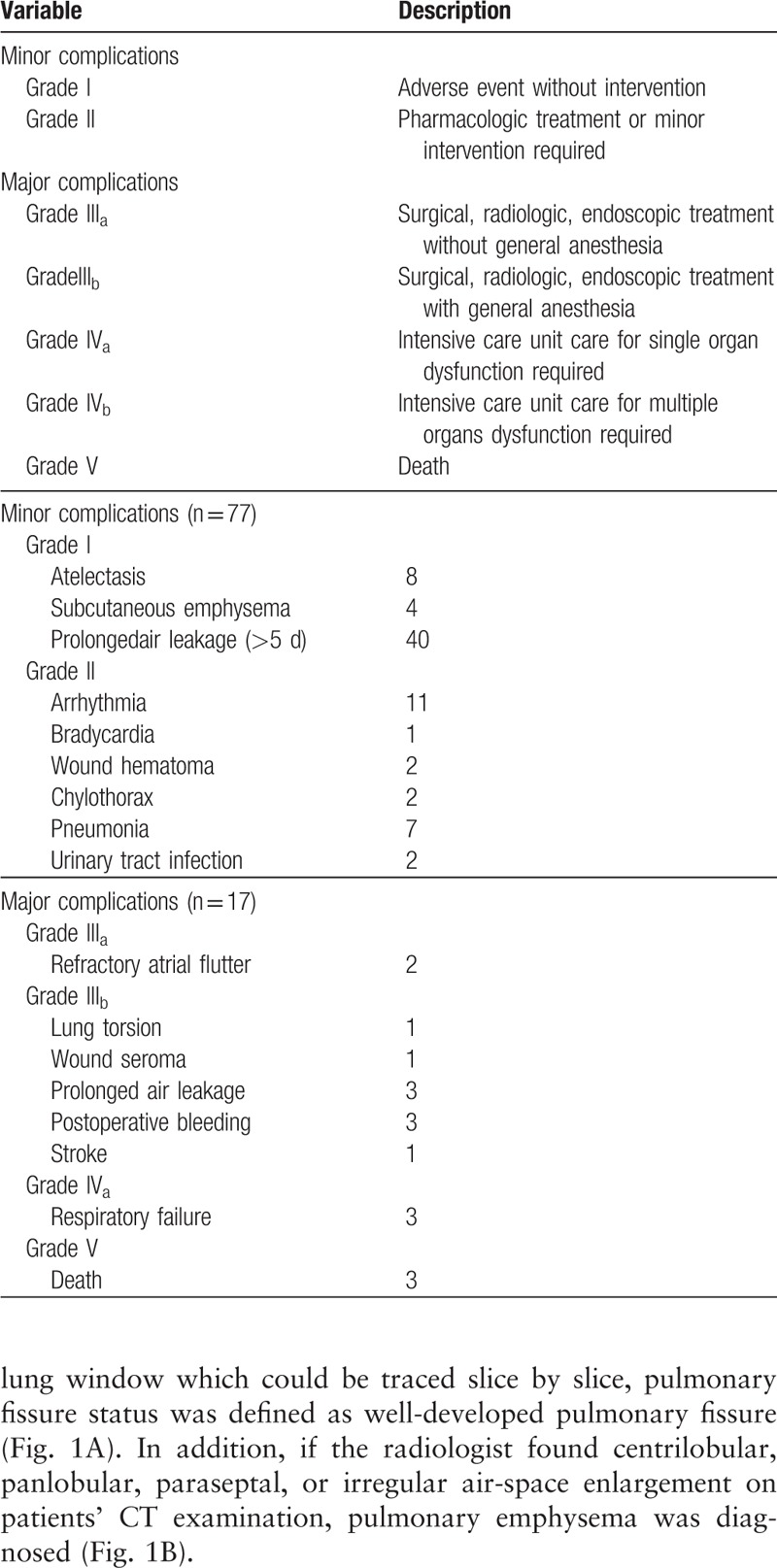
Clavien-Dindo classification of surgical complications and 90-day postoperative complications.

### 2.4 Pulmonary fissure development and emphysema evaluation

**Figure 1:**
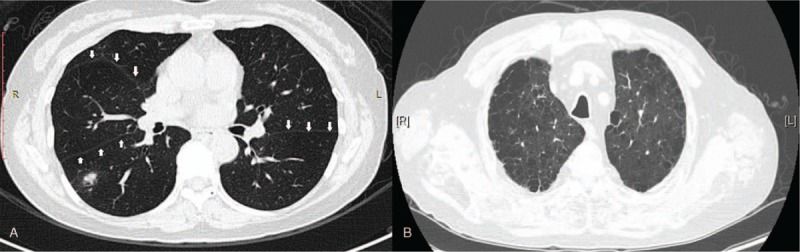
(A) Well-developed pulmonary fissure: Tracing pulmonary fissure development slice by slice. (B) Pulmonary emphysema: centrilobular and paraseptal subtypes.

Before surgery, each patient received a chest CT. We reviewed each enrolled patients’ CT. If there was a clear fissure line in the lung window which could be traced slice by slice, pulmonary fissure status was defined as well-developed pulmonary fissure (Fig. 1A). In addition, if the radiologist found centrilobular, panlobular, paraseptal, or irregular air-space enlargement on patients’ CT examination, pulmonary emphysema was diagnosed (Fig. 1B).

### 2.5 Follow-up

The clinical surveillance program depended on the disease characteristics. Patients with malignant diseases returned to the outpatient clinics within a 3-month interval for first 2 years, then every 6 months annually up to 5 years. Those with benign disease only returned to the outpatient clinics within a 3-month interval for the first year.

### 2.6 Statistical analysis

All data were collected retrospectively based on the hospital information system. A descriptive quantitative and qualitative assessment of morbidity and mortality was carried out. Continuous variables were expressed as mean ± standard deviation. Categorical variables and continuous variables were tested by Fisher exact test and Student *t* test respectively. Odds ratios (ORs) were calculated for risk factors for the presence of complications. *P* values smaller than .05 were considered significant. All analyses were performed using the SPSS (Version 19, Chicago, IL).

## 3 Results

Between January 2014 and February 2017, 442 SPVATS lung anatomic resections were performed in 2 centers, which included 16 pneumonectomy, 21 bilobectomy, 356 lobectomy, and 49 segmentectomy. There were 136 females (30.8%) and 306 males (69.2%). The mean age was 63.94 ± 11.43 years old. Two hundred eighty-three patients (64%) had a history of smoking and 143 (32.4%) were current smokers. The mean forced expiratory volume in 1 second (FEV_1_) was 2.29 ± 0.63. As regards the indication for SPVATS anatomic resections, 364 patients (82.4%) were primary lung cancer, 45 patients (10.2%) were secondary lung cancer, and 33 patients (7.5%) were benign lesions. For primary lung cancer, 57 patients received neoadjuvant chemotherapy. The main tumor locations are summarized in Table 2, whereby the most frequent tumor locations were the right upper lobe (149) and left upper lobe (103).

**Table 2:**
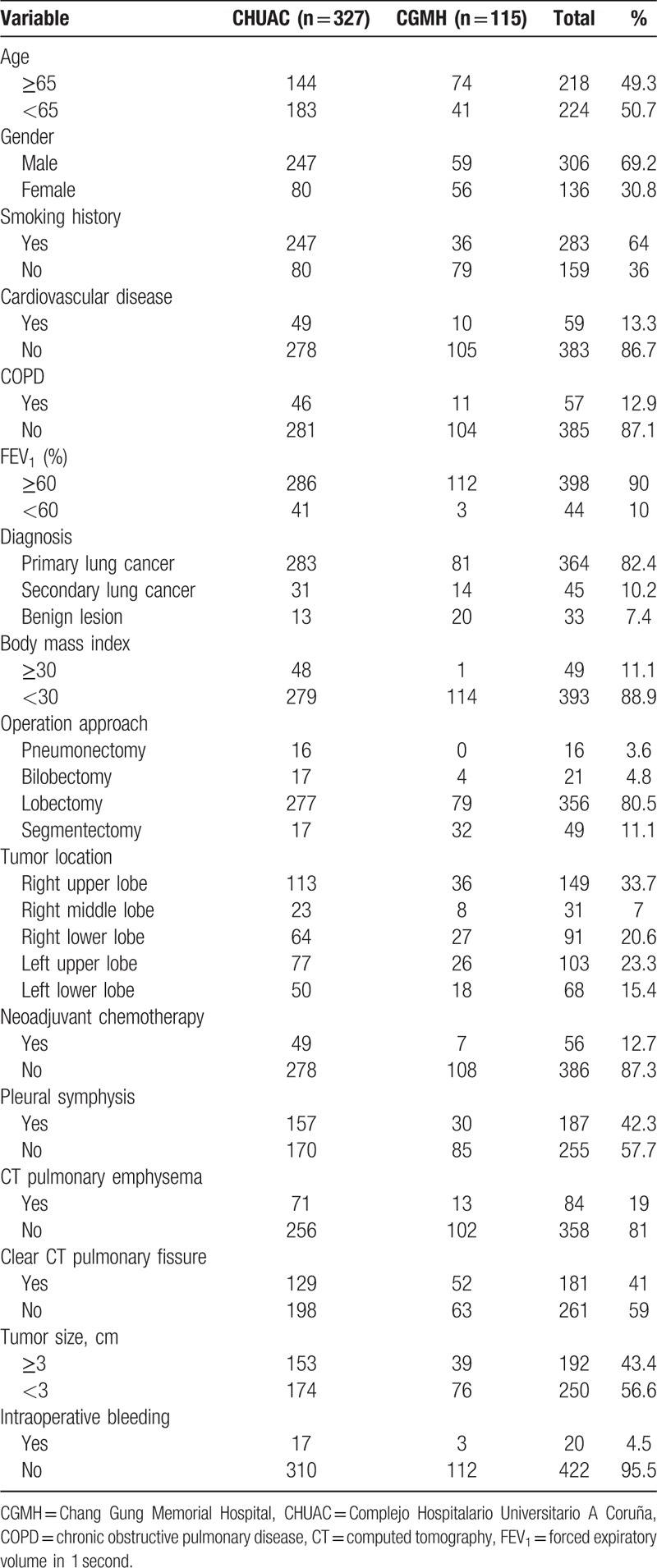
Patient characteristics of 2 centers.

### 3.1 Complications

There was no intraoperative death in our cohort. The mortality rate at 30 and 90 days was 0.7% (3 cases). One patient who had human immunodeficiency virus (HIV) infection suffered from thrombocytopenia, bradycardia, and died on postoperation day 2. The second patient developed acute respiratory failure with refractory hypoxemia on postoperation day 10 and died on postoperation day 19. The third patient, who had had a heart transplant 10 years prior, developed pneumonia on postoperation day 8 and died on postoperation day 17 due to severe sepsis. A total of 94 patients (21.3%) had complications. There were 17 major complications (3.9%): 2 cases required reintubation due to respiratory failure, 12 cases required reintervention due to severe air leakage and subcutaneous emphysema, postoperative bleeding, refractory atrial flutter, or wound seroma. Three cases died within the postoperative 90 days as mentioned above. Minor complications occurred in 77 patients (17.4%), whereby the majority of minor complications were PAL (>5 days) and postoperative arrhythmia. A detailed list of complications is reported in Table 1. As regards the different operation methods, there was no significant difference between segmentectomy (6/49, 12.2%), lobectomy (78/356, 22%), bilobectomy (7/21, 33.3%), and pneumonectomy (5/16, 31.3%, *P* = .164). The mean chest tube duration for all patients was 3.96 days (range 1–23 days), and the overall mean postoperative stay was 5.28 days (range 2–30 days).

On univariate risk factor analysis, male gender was a statistically significant risk factor for postoperative complications (26.4% vs 11%, *P* < .001). In patients with ischemia heart disease, the overall complication rate was 32.2%, which was significantly higher than in those patients without ischemia heart disease (20.1%, *P* = .036). There were fewer complications in patients without chronic obstructive pulmonary disease (COPD) than in those with COPD (19.0% vs 39.6%, *P* < .001). Furthermore, we observed that patients with a smoking history, including active smokers and previous smokers, had a higher likelihood of postoperative complications (25.4% vs 15.0%, *P* = .011). Preoperative induction therapy did not significantly associate with postoperative complications (*P* = .093), drainage duration (*P* = .259), and postoperative hospital stay (*P* = .628). CT fissure line was also a significant postoperative complications predictor. Patients with a clear CT fissure line were less likely to suffer from postoperative complications (15.4% vs 26.0%, *P* = .008). Pleural symphysis was also an important complications prognostic factor. Patients with pleural symphysis had a higher postoperative complications rate (31.5% vs 14.5%, *P* < .001). Tumor size ≥3 cm also meant high probability of postoperative complications. In addition, we also found significantly different postoperative complication rates in the 2 hospitals (*P* = .002).

On multivariate analysis, pleural symphysis, pulmonary emphysema, well-developed pulmonary CT fissure line, and tumor size ≥3 cm remained predictors of postoperative complications (Table 3).

**Table 3:**
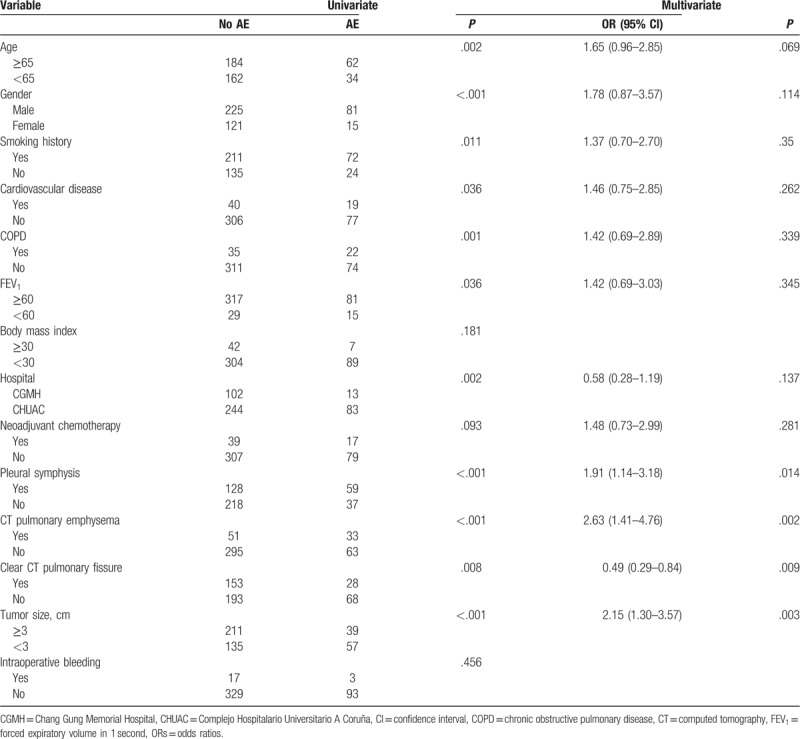
Predictors for adverse events (AE) after SPVATS anatomic resection.

## 4 Conclusion

In recent years, minimally invasive surgery for lung anatomic resections has gradually become accepted all over the world. The expected benefits of VATS anatomic resection include less pain, immune suppression and complications, shorter hospital stay and faster patient recovery.^[13–17]^ Compared with traditional VATS, single-port VATS is like a child exploring all kinds of possibilities. Every type, from minor and intermediate procedures to more complex procedures has been described over the past few years.^[7,10,18]^ Although a series of retrospective studies has shown that single-port VATS is superior to the traditional VATS approach in terms of early outcomes^[6,7,19]^ (i.e., postoperative pain, chest tube drainage, and hospital stay) and have emphasized the feasibility and safety of this procedure, there has always been some skepticism about treatment and oncological results. Preliminary reports on single-port VATS have led to many discussions and eliminated some of the doubts of opponents,^[6–9]^ but suspicions or criticism regarding the potential compromise of oncological results or patients’ postoperative morbidity and mortality still remained. Postoperative complications have always been a serious concern regarding patients’ safety and the potential postoperative occurrence of catastrophic events. Data from the society of thoracic surgeons general thoracic surgery database showed a 32% morbidity rate and 2% 30-day mortality rate in 5957 open thoracotomy lobectomy cases. In another large series of open lobectomies, morbidity ranged from 28% to 38% and mortality ranged from 1.2% to 2.9%.^[20]^ In recent large national database analysis in the United States,^[21]^ VATS or robotically assisted lobectomy showed favorable morbidity (45.3%, 43.8%) and mortality (2.6%, 1.2%) results when compared with open surgery (54.1%, 0.3%). However, the literature on single-port VATS associated postoperative complications was scant and lacked complete analysis. Our initial results made up for the deficiency in this area with acceptable 30-day mortality (0.7%) and major morbidity (3.9%). Moreover, our results correlated with other large VATS series reports, for example, McKenna et al^[22]^ reported the largest single-institution series of VATS lobectomies with a 0.8% mortality and 15.3% morbidity rate (1100 cases). Onaitis et al^[23]^ showed that VATS lobectomy could be safely applied to a spectrum of malignant and benign pulmonary diseases associated with a 1% mortality and 23.2% morbidity rate (500 cases). It seemed that SPVATS anatomic resection would not compromise patient safety or increase related postoperative complications just by reducing port numbers.

Prolonged air leak (PAL) was the most common postoperative complication in our cohort. Although there was no clear consensus on the duration of PAL, which usually lasted longer than 5 or 7 days postoperatively, we used the rigorous PAL definition (PAL > 5 days) in our study. By risk factor analysis, we found SPVATS postoperative complications were associated independently with pleural symphysis, pulmonary emphysema, unclear CT pulmonary fissure line, and tumor ≥3 cm. For those surgeons who want to attempt SPVATS major lung resection, those factors could be considered significant indices to support the choice of suitable surgical candidates. In addition, PAL might be significantly reduced by avoiding fissure dissection and initiating anatomic resection from hilar structures and completing pulmonary fissure at the last step. With a well-developed pulmonary fissure, the SPVATS surgeon can easily dissect following a correct surgical plan without lung parenchyma injury. Previously, Lee et al^[24]^ proposed a classification system for pulmonary fissures based on the degree of fissure development and the extent of exposure of the pulmonary artery. With the help of imaging tools, we could classify patients into different groups according to the extent of pulmonary fissure development so that we could simultaneously attend to technique training and patient safety. However, due to a lack of records of real visual inspection of pulmonary fissure development in this retrospective study, further evaluation is warranted.

Neoadjuvant induction therapy might lead to tissue adhesion, an indistinct interface and increased vascular fragility, and it will have a great impact on patients’ ability to recovery. The incidence of surgical complications after induction therapy has been reported to be as high as 9.5% to 43.5%.^[25–27]^ In the present study, the postoperative complication rate did not show a statically significant difference between patients with/without induction therapy (30% vs 20.3%, *P* = .093). To date, only a few studies have conducted detailed analysis of SPVATS anatomic resection in patients who underwent induction therapy. Even compared with results of thoracotomy or VATS anatomic resection in this kind of patients, our study showed results consistent with previous studies. Yang et al^[26]^ evaluated the postoperative complication rate of 272 locally advanced patients (thoracotomy: 203, VATS: 69) and reported their complication rate was 48% and 41%, respectively. Huang et al^[27]^ evaluated the outcomes of 43 patients with stage IIA–IIIB Non small cell lung cancer (NSCLC) patients who received induction chemotherapy followed by VATS resections and reported 9.5% complication rate.

This research has several limitations. First, although the present study involved a relatively large series of SPVAT anatomic resections with postoperative complications, this was a retrospective review and inevitably had downsides to its neutrality and confounders for which we cannot account. For example, there was no consensus about how many SPVATS cases constituted enough experience for a surgeon to be familiar with such new surgical techniques. The impact on patients of the surgeon's learning curve for any new procedure might be longer operation time and higher postoperative complication rate. Individual differences between surgeons and the lack of objective assessment to evaluate each surgeon's SPVATS techniques were potential confounding factors in our cohort. Second, different surgical approaches might produce different postoperative complication rates. Shapiro et al^[28]^ reported that pneumonectomy was associated with a significant incidence of perioperative morbidity (30.4%) and mortality (5.6%) in the national database. According to a previous report, the overall morbidity for bilobectomy is around 21.1% to 45.8%.^[29,30]^ Larger discrepancies between the size of the pleural cavity and the remnant structures are considered one of the main causes of morbidity. In our cohort, there was no significant difference between different approaches, which may be the result of limited numbers of enrolled patients receiving bilobectomy and pneumonectomy. Third, since the lack of unified CT slice thickness and real inspection data of pulmonary fissure development were also confounders in our study, a more rigorous investigation is needed. Fourth, different patient populations with unequal case numbers between the 2 hospitals constituted another limitation to this study. For instance, postoperative complication rate was significantly different between CHUAC and CGMH in univariate analysis. But, after multivariate analysis, there was no difference between the 2 hospitals. We believe that these limitations were favorably compensated by the other characteristics. Finally, the inherent differences in the diagnosis also had a varying degree of impact on postoperative complications. Limited by the case numbers, it was difficult for us to tell the difference between malignant and benign disease and do further subgroup analysis to investigate this problem.

To conclude, the current results revealed several clinical factors that may be useful predictors for predicting postoperative complications in patients with disease following SPVATS anatomic resections. Surgeons should therefore exercise additional caution in patients with these risk factors before surgery.

## Author Contributions

**Study conception and design**: Yung Chia Kuo, Ching Feng Wu, Diego Gonzalez-Rivas, de la Torre Mercedes, Ricardo Fernandez, Ching Yang Wu.

**Acquisition of data**: Ching Feng Wu, de la Torre Mercedes, Ricardo Fernandez, Maria Delgado, Eva Fieira, Ching Yang Wu, Ming Ju Hsieh, Marina Paradela, Yin Kai Chao, Diego Gonzalez-Rivas.

**Analysis and interpretation of data**: Ching Feng Wu, Maria Delgado, Ching Yang Wu.

**Drafting of manuscript**: Yung Chia Kuo, Ching Feng Wu, de la Torre Mercedes, Ricardo Fernandez, Diego Gonzalez-Rivas.

**Critical revision**: Ching Feng Wu, de la Torre Mercedes, Diego Gonzalez-Rivas.

**Data curation:** Yung Chia Kuo, Ching Yang Wu, Maria Delgado, de la Torre Mercedes, Ricardo Fernandez, Eva Fieira, Ming Ju Hsieh, Marina Paradela, Yin Kai Chao, Ching-Feng Wu.

**Writing—original draft:** Yung Chia Kuo.

**Conceptualization:** Diego Gonzalez-Rivas, Ching-Feng Wu.

**Formal analysis:** Diego Gonzalez-Rivas, Ching Yang Wu, de la Torre Mercedes, Ricardo Fernandez, Ming Ju Hsieh, Ching-Feng Wu.

**Investigation:** Diego Gonzalez-Rivas, de la Torre Mercedes.

**Supervision:** Diego Gonzalez-Rivas, Ching Yang Wu, Ricardo Fernandez, Eva Fieira, Ming Ju Hsieh, Marina Paradela, Yin Kai Chao, Ching-Feng Wu.

**Validation:** Diego Gonzalez-Rivas, Maria Delgado, de la Torre Mercedes, Ricardo Fernandez, Eva Fieira, Marina Paradela, Yin Kai Chao, Ching-Feng Wu.

**Writing—review & editing:** Diego Gonzalez-Rivas, Ching-Feng Wu.

**Methodology:** Maria Delgado.

**Resources:** Maria Delgado.

**Project administration:** Eva Fieira.

**Software:** Yin Kai Chao.

## References

[1] Roviaro G Rebuffat C Varoli F Videoendoscopic pulmonary lobectomy for cancer. Surg Laparosc Endosc 1992;2:244–7. 1341539

[2] McKenna RJ Jr Lobectomy by video-assisted thoracic surgery with mediastinal node sampling for lung cancer. J Thorac Cardiovasc Surg 1994;107:879–81. 8127117

[3] Gonzalez D Paradela M Garcia J Single-port video-assisted thoracoscopic lobectomy. Interact Cardiovasc Thorac Surg 2011;12:514–5. 21131682 10.1510/icvts.2010.256222

[4] Gonzalez-Rivas D Paradela M Fieira E Single-incision video-assisted thoracoscopic lobectomy: initial results. J Thorac Cardiovasc Surg 2012;143:745–7. 21868042 10.1016/j.jtcvs.2011.07.049

[5] Tamura M Shimizu Y Hashizume Y Pain following thoracoscopic surgery: retrospective analysis between single-incision and three-port video-assisted thoracoscopic surgery. J Cardiothorac Surg 2013;8:153. 23759173 10.1186/1749-8090-8-153 PMC3691684

[6] Shen Y Wang H Feng M Single- versus multiple-port thoracoscopic lobectomy for lung cancer: a propensity-matched study. Eur J Cardiothorac Surg 2016;49 (suppl 1):i48–53. 26464451 10.1093/ejcts/ezv358

[7] Wu CF Gonzalez-Rivas D Wen CT Comparative short-term clinical outcomes of mediastinum tumor excision performed by conventional VATS and single-port VATS: is it worthwhile? Medicine (Baltimore) 2015;94:e1975. 26559275 10.1097/MD.0000000000001975 PMC4912269

[8] Hsu PK Lin WC Chang YC Multiinstitutional analysis of single-port video-assisted thoracoscopic anatomical resection for primary lung cancer. Ann Thorac Surg 2015;99:1739–44. 25827674 10.1016/j.athoracsur.2015.01.041

[9] Liu CY Cheng CT Wang BY Number of retrieved lymph nodes and postoperative pain in single-incision and multiple-incision thoracoscopic surgery. Ann Surg 2017;265:E76–7. 28486293 10.1097/SLA.0000000000001320

[10] Gonzalez-Rivas D Paradela M Fernandez R Uniportal video-assisted thoracoscopic lobectomy: two years of experience. Ann Thorac Surg 2013;95:426–32. 23219257 10.1016/j.athoracsur.2012.10.070

[11] Dindo D Demartines N Clavien PA Classification of surgical complications: a new proposal with evaluation in a cohort of 6336 patients and results of a survey. Ann Surg 2004;240:205–13. 15273542 10.1097/01.sla.0000133083.54934.ae PMC1360123

[12] Brunelli A Drosos P Dinesh P The severity of complications is associated with postoperative costs after lung resection. Ann Thorac Surg 2017;103:1641–6. 28189276 10.1016/j.athoracsur.2016.10.061

[13] Nicastri DG Wisnivesky JP Litle VR Thoracoscopic lobectomy: report on safety, discharge independence, pain, and chemotherapy tolerance. J Thorac Cardiovasc Surg 2008;135:642–7. 18329487 10.1016/j.jtcvs.2007.09.014

[14] Fang HY Chen CY Wang YC Consistently lower narcotics consumption after video-assisted thoracoscopic surgery for early stage non-small cell lung cancer when compared to open surgery: a one-year follow-up study. Eur J Cardiothorac Surg 2013;43:783–6. 22864790 10.1093/ejcts/ezs370

[15] Craig SR Leaver HA Yap PL Acute phase responses following minimal access and conventional thoracic surgery. Eur J Cardiothorac Surg 2001;20:455–63. 11509263 10.1016/s1010-7940(01)00841-7

[16] Yang CF Sun Z Speicher PJ Use and outcomes of minimally invasive lobectomy for stage I non-small cell lung cancer in the National Cancer Data Base. Ann Thorac Surg 2016;101:1037–42. 26822346 10.1016/j.athoracsur.2015.11.018 PMC4763985

[17] Nwogu CE D’Cunha J Pang H VATS lobectomy has better perioperative outcomes than open lobectomy: CALGB 31001, an ancillary analysis of CALGB 140202 (Alliance). Ann Thorac Surg 2015;99:399–405. 25499481 10.1016/j.athoracsur.2014.09.018 PMC5616174

[18] Rocco G Martucci N La Manna C Ten-year experience on 644 patients undergoing single-port (uniportal) video-assisted thoracoscopic surgery. Ann Thorac Surg 2013;96:434–8. 23790399 10.1016/j.athoracsur.2013.04.044

[19] Mu JW Gao SG Xue Q A propensity matched comparison of effects between video assisted thoracoscopic single-port, two-port and three-port pulmonary resection on lung cancer. J Thorac Dis 2016;8:1469–76. 27499933 10.21037/jtd.2016.05.64 PMC4958795

[20] Boffa DJ Allen MS Grab JD Data from The Society of Thoracic Surgeons General Thoracic Surgery database: the surgical management of primary lung tumors. J Thorac Cardiovasc Surg 2008;135:247–54. 18242243 10.1016/j.jtcvs.2007.07.060

[21] Kent M Wang T Whyte R Open, video-assisted thoracic surgery, and robotic lobectomy: review of a national database. Ann Thorac Surg 2014;97:236–42. 24090577 10.1016/j.athoracsur.2013.07.117

[22] McKenna RJ Jr Houck W Fuller CB Video-assisted thoracic surgery lobectomy: experience with 1,100 cases. Ann Thorac Surg 2006;81:421–5. 16427825 10.1016/j.athoracsur.2005.07.078

[23] Onaitis MW Petersen RP Balderson Thoracoscopic lobectomy is a safe and versatile procedure: experience with 500 consecutive patients. Ann Surg 2006;244:420–5. 16926568 10.1097/01.sla.0000234892.79056.63 PMC1856541

[24] Lee S Lee JG Lee CY Pulmonary fissure development is a prognostic factor for patients with resected stage I lung adenocarcinoma. J Surg Oncol 2016;114:848–52. 27633283 10.1002/jso.24438

[25] Fujita S Katakami N Takahashi Y Postoperative complications after induction chemoradiotherapy in patients with non-small-cell lung cancer. Eur J Cardiothorac Surg 2006;29:896–901. 16675259 10.1016/j.ejcts.2006.03.023

[26] Yang C-FJ Meyerhoff RR Mayne NR Long-term survival following open versus thoracoscopic lobectomy after preoperative chemotherapy for non-small cell lung cancer. Eur J Cardiothorac Surg 2016;49:1615–23. 26719408 10.1093/ejcts/ezv428 PMC4867396

[27] Huang J Xu X Chen H Feasibility of complete video-assisted thoracoscopic surgery following neoadjuvant therapy for locally advanced non-small cell lung cancer. J Thorac Dis 2013;5 (suppl 3):S267–73. 24040535 10.3978/j.issn.2072-1439.2013.08.24 PMC3771588

[28] Shapiro M Swanson SJ Wright CD Predictors of major morbidity and mortality after pneumonectomy utilizing the Society for Thoracic Surgeons General Thoracic Surgery Database. Ann Thorac Surg 2010;90:927–34. 20732520 10.1016/j.athoracsur.2010.05.041

[29] Gómez MT Jiménez MF Aranda JL The risk of bilobectomy compared with lobectomy: a retrospective analysis of a series of matched cases and controls. Eur J Cardiothorac Surg 2014;46:72–5. 24242849 10.1093/ejcts/ezt521

[30] Thomas PA Falcoz P-E Bernard A Bilobectomy for lung cancer: contemporary national early morbidity and mortality outcomes. Eur J Cardiothorac Surg 2016;49:e38–43. 27070154 10.1093/ejcts/ezv407

